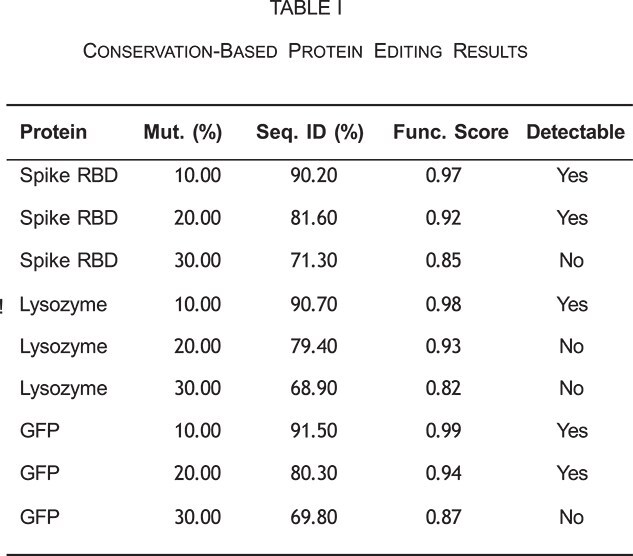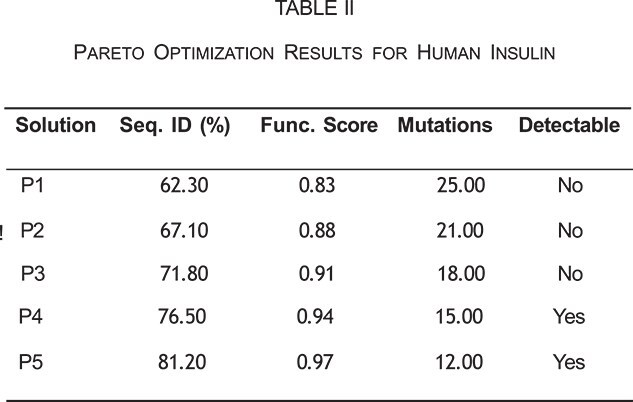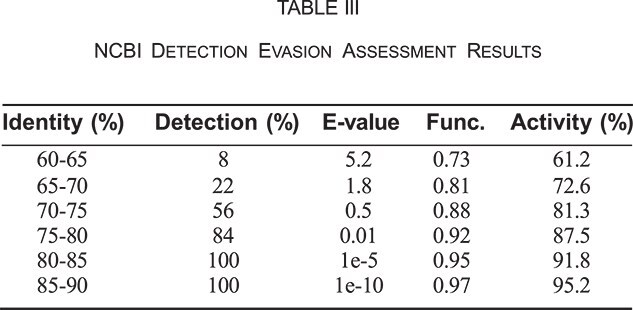# Function-aware biosecurity screening: datasets, benchmarks, and detection systems for next-generation pathogen identification

**DOI:** 10.1093/bib/bbaf631.044

**Published:** 2025-12-12

**Authors:** Emilin Mathew

**Affiliations:** Stanford University, Stanford, CA 94305, United States emilin@stanford.edu

## Abstract

**Aim:**

The proliferation of synthetic biology tools and artificial intelligence has intensified concerns about the misuse of biological agents, making robust biosecurity screening systems increasingly critical. Traditional screening frameworks rely on homology based methods, which flag sequences by similarity to known pathogens. These approaches frequently degrade on de novo or engineered sequences that retain pathogenic function without recognizable identity. This work presents three complementary solutions that advance function- aware screening.

**Methods:**

OmniTox is a contemporary benchmark for binary toxin versus non-toxin protein classification that synthesizes vetted public classification into a single dataset. The dataset construction pipeline employs stringent filtering criteria ap- plied uniformly to both positive and negative classes. Toxin sequences (positive class) are sourced from UniProtKB using the standardized toxin keyword (KW-0800), with sequences constrained to 6–1024 amino acids to exclude peptide fragments and unusually large proteins that may represent annotation artifacts. Both archaeal and viral sequences are excluded to focus on bacterial and eukaryotic toxins relevant to biosecurity applications. Non-toxin sequences (negative class) are drawn exclusively from Swiss-Prot reviewed entries, ensuring high- quality annotations and reducing noise from computationally predicted proteins.

The curation process incorporates comprehensive dedupli- cation and overlap detection. All sequences are checked for exact matches and high-similarity overlaps between positive and negative classes to prevent data leakage. Additionally, metadata integration provides rich contextual information including organism taxonomy, protein length, and UniProt accession numbers, enabling downstream analyses of model performance across different biological contexts. By consolidating frag- mented sources into a clean benchmark, OmniTox provides a stable foundation for developing and evaluating biosecurity screening systems in both research and provider settings.

OmniVariant provides a computational framework for gen- erating synthetic pathogenic variants to train and evaluate next-generation biosecurity screening systems. Traditional homology-based screening approaches fail when confronted with engineered sequences that retain pathogenic function while evading sequence similarity detection. OmniVariant addresses this vulnerability by systematically generating functional protein variants that challenge existing detection paradigms.

The framework employs conservation-guided mutagenesis integrated with multi-objective Pareto optimization to preserve functionally critical residues while systematically reducing sequence homology to reference databases. Conservation analysis utilizes ESM-2 protein language model embeddings to identify functional regions without requiring structural data, making the approach broadly applicable across diverse protein families. A conservation threshold of 0.7 effectively identifies catalytic sites, binding domains, and other functionally essential regions across multiple protein classes.

Computational validation was conducted using representative proteins from different functional classes, including viral proteins (SARS-CoV-2 Spike RBD), antimicrobial enzymes (human lysozyme), and reporter proteins (GFP). The frame- work’s performance was evaluated across multiple mutation rates to establish optimal parameters for function preservation while achieving detection evasion.

**Results:**

Results demonstrate that mutation rates of approximately 30% consistently produce variants below NCBI detection thresholds while maintaining function scores above 0.80.

While conservation-guided mutagenesis provides a founda- tion for function preservation, the optimal balance between detectability and function requires multi-objective optimization. Using human insulin as a test case, Pareto optimization identified optimal solutions that balance sequence divergence with function retention, consistently outperforming approaches that optimize only sequence identity or function independently. The Pareto front reveals a critical trade-off region around 70–75% sequence identity, where variants can evade detection while maintaining high functional scores above 0.90.

To evaluate the framework’s practical utility, an assessment was conducted to determine the detection threshold of standard biosecurity screening systems. Fifty variants of beta-lactamase were generated with varying degrees of sequence divergence (60–90% identity) and submitted to NCBI BLAST with default parameters to assess detection rates, E-values, and functional predictions.

The detection threshold occurs around 70–75% sequence identity for most proteins, establishing a critical boundary for evasion capabilities. Variants in the 65–70% identity range achieved optimal balance with low detection rates (22%), maintained good function scores (0.81), and preserved 72.6% of original catalytic activity. The framework successfully identified mutations that maximize divergence in non-essential regions while preserving core functional elements.

These results demonstrate that OmniVariant successfully generates protein variants that maintain substantial biological function (function scores *>*0.80) while achieving sequence identities below 70%, effectively evading standard homology-based detection systems. The framework provides critical training benchmarks for developing more robust function-aware biosecurity screening technologies.

Building on these foundations, Omnyra is a function-based pathogen screening system that leverages protein language models and specialized classifiers to assess functional risk independent of sequence homology. The system employs deep learning architectures to identify pathogenic potential through learned functional representations rather than traditional sequence similarity metrics. Omnyra addresses two critical operational scenarios: (i) novel or engineered sequences that evade homology-based detection while retaining pathogenic function, and (ii) ambiguous detections requiring enhanced interpretability for expert review. By incorporating functional embeddings and attention mechanisms, the system provides both classification confidence scores and feature attribution maps to support evidence-based screening decisions in high- stakes biosecurity contexts.

Together, these three complementary tools establish a comprehensive foundation for advancing biosecurity screening from homology-based detection toward robust, function-aware systems capable of identifying threats regardless of sequence engineering.

**References:**

1. Wittmann B.J., Alexanian T., Bartling C., Beal J., Clore A., Diggans J., Flyangolts K., Gemler B.T., Mitchell T., Murphy S.T., Wheeler N.E., Horvitz E. ‘Toward AI-resilient screening of nucleic acid synthesis orders: Process, results, and recom- mendations.’ *bioRxiv* 2024; doi:10.1101/2024.12.02.626439.

2. Challacombe C.A., Haas N.S. ‘Towards a dataset for state of the art protein toxin classification.’ *bioRxiv* 2024; doi:10.1101/2024.04.14.589430.

3. Zhang Z., Chakraborty S., Bedi A.S., Mathew E., Sar- avanan V., Cong L., Velasquez A., Lin-Gibson S., Blewett M., Hendrycs D., London A.J., Zhong E., Raphael B., Ma J., Xing E., Altman R., Church G., Wang M. ‘Generative AI for biosciences: Emerging threats and roadmap to biosecurity.’ *arXiv* 2025; doi:10.48550/arXiv.2510.15975.